# Lectures on the Principles and Practice of Physic

**Published:** 1858-07

**Authors:** 


ART. V.—*Lectures on the Principles and Practice of Physic.* Delivered at King's College, London. By THOMAS WATSON, M.D., Fellow of the Royal College of Physicians; late Physician to the Middlesex Hospital, etc. Two volumes, 8vo., pp. 1856. Fourth edition, revised. London: John W. Parker & Son. 1858.

IN announcing a fourth edition of Dr. Watson's work we are performing one of those pleasant duties sometimes connected with literary notices. We are welcoming back an old and revered friend, whose imperative obligations have kept him from us for a few years, but who comes back with his wisdom extended, his liveliness and genial kindness the same, to charm us and enlighten us as of old.

Dr. Watson's lectures, which common consent has made the text-book of the profession in this country as well as in England, have been for several years out of print. The unceasing toil of busy professional life had deprived him of the leisure necessary for their revision, and we have to thank him that he has taken his moments of rest, not only to re-edit, but also to extend them, and to incorporate whatever recent investigations have added to the science.

Of new matter we notice especially an alteration in the lectures on hypertrophy and on transformation of tissue, conformably with the results obtained by Mr. Paget; remarks on fatty degeneration of the heart, rendering a good account of Dr. Quain's researches; references to the views of Brown-Séquard concerning the spinal cord; to those of Kirkes, in connection with obstruction of the arteries by vegetations washed into them; to the labors of Garrod on the chemistry of scurvy and gout; of Frerich's and others concerning Bright's disease; and many additions on cardiac and abdominal affections.

In the first volume there is a very interesting statement regarding the application of anæsthetics. Dr. Watson, without wishing to detract from "the triumph and glory" of the discovery, gives us some singular information, which proves how closely a great discovery may be approached, and yet missed. A patient of his told him this story of herself:—

> "She had been sorely tried, in her early years, with paroxysms of urgent dyspnœa, frequently occurring; and her life was thought to be in danger. After fruitless trials of various other remedies, the following method was adopted, with the happiest results, under the advice of a physician of high promise, who died young, the late Dr. Woolcombe, of Plymouth.
>
> "About two teaspoonfuls of sulphuric æther were poured into a saucer, which was placed on her lap, and over which she breathed, as she sat gasping in bed, with a shawl thrown over her head to prevent the escape of the vapor. Very soon a delightful sensation of tranquillity ensued; she felt (I quote her own words) 'as if going to heaven in the most heavenly way,' and presently she sank back unconscious. As soon as this happened, her husband, by whom the process was managed, withdrew the shawl, and in a short time Lady Martin gradually recovered, breathing calmly. This mode of quieting her attacks of asthma was begun in 1806, a few years after the publication of Sir Humphrey Davy's hint; and it was repeated again and again, sometimes twice in the same day, for a very considerable period."

The note appended to this case is very curious. Dr. Watson tells us that the practice of benumbing the sensibility of a patient about to undergo a surgical operation, by means of inhaling vapors, may be traced back to the twelfth century, as was ascertained from the pages of an Italian work in the British Museum. One Hugo da Lucca bequeathed to his son the secret of making several chemical preparations, which the latter, who finally became Bishop of Cervia, published. Among them was a most potent caustic and a soporific, which, by being inhaled, possessed the power of putting the patient to sleep before undergoing a painful operation, ("*ed un soporifero che per mezzo del solo odorato assopiva i malati, in occasione di operazioni dolorose, che dovessero soffire.*") That the remedy prepared from the juice of various anodynes was simply used by inhalation, is undoubted; the formula for its preparation proves it.*

At the end of the lecture on blood-letting is another note which will attract much attention. It is an answer to the arguments of Dr. Bennett against the use of blood-letting and antiphlogistics; an answer the fairness of which is only equalled by its courtesy and the language in which it is clothed. Dr. Bennett takes the ground that antiphlogistics are not necessary, and that their relative abandonment, especially that of blood-letting, is due to the advance of our knowledge of pathology and diagnosis. Dr. Watson testifies, from the experience in his own person, to the efficacy of bleeding and to its power of cutting short disease. He is persuaded that we bleed less because diseases have changed their type, and that the argument against venæsection, that it deteriorates the blood, is not a good one, since in inflammation there is reason to believe that the inflamed part spreads infection through the blood; this we prevent if we can arrest the inflammation. Statistics ought not to be permitted to determine our practice:

"To use or to withhold a given remedy simply because it is found, by numerical calculation, that in cases nominally the same recoveries have been more frequent when that remedy was employed on the one hand, or omitted on the other, would be to sacrifice the plain and perhaps pressing indications of a particular case to the statistical averages of diseases having merely a common denomination."

In the chapter on mercurial poisoning, Dr. Watson mentions the view of M. Melsens as to the actual union of the metallic substance with the affected part. The trembling occasioned by the mercury is best removed by small doses of strychnia, by iron and by quinia; while the poison may be expelled by preparations of iodine. In iron he places most faith.

The lectures on fever show, however, most change. Dr. Watson frankly avows that he no longer thinks that a definite distinction between the forms of continued fever does not exist. "The Dr. Jenner of our time, with patience and sagacity worthy of the great name he bears," has proved to the English profession that the typhus and typhoid fever are not the same; and this view Dr. Watson adopts. He remarks, that Dr. Bartlett, in 1842, had testified to the same distinction in the fevers of the United

* "Haec omnia in unum commisce in vase æneo: ac deinde in istud mitte spongiam novam, quod totum ebulliat: et tamdiu ad solem canicularibus diebus donec omnia consumat: et decoquatur in ea: quoties autem opus erit, mittas ipsam spongiam in aquam calidam per unam horam: et naribus apponatur; quousque somnum capiat: qui incidendus est: et sic fiat chirurgia: qua peracta, ut excitetur: aliam spongiam in aceto infusam, frequenter ad nares ponas."

States. He might have added, that before the year 1837, Dr. Gerhard had proved by accurate observation the identity of our typhoid fever with that described by Louis, and had drawn the difference between it and typhus.

Addison's disease also receives its full share of consideration, and a case is added which came under Dr. Watson's care. The patient had a tanned appearance, his hands were bronzy, with pink-contrasting finger nails. The course of the disease was marked by fluctuations: he regained at one time flesh and strength, and the bronzed hue varied in depth, but he finally sank under an acute laryngeal affection. The supra-renal capsules were found to be enlarged and infiltrated with crude and suppurating tubercular matter.

The lectures on other abdominal diseases have also been somewhat extended.

All the additions in these volumes are in the same masterly style, and show the same clear erudition and high reasoning powers with which we are familiar from former editions. In his introductory chapter Dr. Watson paraphrases a passage from Bacon:—"The lecturer must not be the ant, collecting all things indiscriminately from all quarters; nor the spider, seeking no materials abroad, but spinning his web of speculative doctrine from within himself; but rather the bee, extracting crude honey from various flowers, storing it up in the recesses of his brain, and submitting it to the operation of his internal faculties until it be matured and ready for use." Could any one be found who might serve better as the original of this sketch of a model lecturer than Dr. Watson himself? If he had been sitting for his portrait, could it have been more fitly and accurately drawn? Yet there is one point needed to make it complete,—it is to add that the lecturer's skill, his wisdom, his learning, are equalled by the ease of his graceful diction, his eloquence, and the far higher qualities of candor, of courtesy, of modesty, and of generous appreciation of merit in others. May he long remain to instruct us, and to enjoy in the glorious sunset of his declining years the honors, the confidence and love gained during his useful life!

